# Functional diversity of c-di-GMP receptors in prokaryotic and eukaryotic systems

**DOI:** 10.1186/s12964-023-01263-5

**Published:** 2023-09-25

**Authors:** Fazlurrahman Khan, Geum-Jae Jeong, Nazia Tabassum, Young-Mog Kim

**Affiliations:** 1 Marine Integrated Biomedical Technology Center, The National Key Research Institutes in Universities, Pukyong National University, Busan, 48513 Republic of Korea; 2 Research Center for Marine Integrated Bionics Technology, Pukyong National University, Busan, 48513 Republic of Korea; 3 Department of Food Science and Technology, Pukyong National University, Busan, 48513 Republic of Korea

**Keywords:** c-di-GMP, Receptor, Bacteria, Interspecies, Eukaryotes, Binding affinity, Evolutionary relatedness

## Abstract

Cyclic bis-(3', 5')-dimeric guanosine monophosphate (c-di-GMP) is ubiquitous in many bacterial species, where it functions as a nucleotide-based secondary messenger and is a vital regulator of numerous biological processes. Due to its ubiquity, most bacterial species possess a wide range of downstream receptors that has a binding affinity to c-di-GMP and elicit output responses. In eukaryotes, several enzymes and riboswitches operate as receptors that interact with c-di-GMP and transduce cellular or environmental signals. This review examines the functional variety of receptors in prokaryotic and eukaryotic systems that exhibit distinct biological responses after interacting with c-di-GMP. Evolutionary relationships and similarities in distance among the c-di-GMP receptors in various bacterial species were evaluated to understand their specificities. Furthermore, residues of receptors involved in c-di-GMP binding are summarized. This review facilitates the understanding of how distinct receptors from different origins bind c-di-GMP equally well, yet fulfill diverse biological roles at the interspecies, intraspecies, and interkingdom levels. Furthermore, it also highlights c-di-GMP receptors as potential therapeutic targets, particularly those found in pathogenic microorganisms.

Video Abstract

**Supplementary Information:**

The online version contains supplementary material available at 10.1186/s12964-023-01263-5.

## Introduction

Cyclic bis-(3', 5')-dimeric guanosine monophosphate (c-di-GMP) is a nucleotide-based signaling molecule discovered in 1987 as an effective activator of cellulose synthase in *Gluconacetobacter xylinus* [1]. Since then, c-di-GMP has been ubiquitously reported in bacterial species [2] as a universal secondary messenger that responds to various environmental and cellular cues [3]. c-di-GMP plays regulatory roles in numerous cellular activities, including cell motility, biofilm formation and dispersion, cell division, differentiation, quorum sensing, and virulence [4]. The intracellular concentration of c-di-GMP is controlled by diguanylate cyclases (DGCs) and phosphodiesterases (PDEs), respectively [5]. DGCs (contain GGDEF domain) catalyze c-di-GMP production by consuming two GTP molecules, whereas PDEs (have EAL or HD-GYP domains) catalyze the degradation of c-di-GMP into a pGpG linear nucleotide or two GTP molecules [6]. Signal transduction occurs through c-di-GMP binding to downstream receptors, triggered by sensing environmental or cellular cues [7]. Recent advances in bioinformatics, molecular biology, structural biology, and biochemistry have identified several types of receptors in bacterial species that bind to c-di-GMP signaling molecules and regulate several cellular processes. The PilZ protein, which regulates flagellar motility, was the first c-di-GMP receptor identified [8]. Moreover, PilZ proteins play key roles in cell aggregation by contributing to chemotaxis, biofilm formation, and colonization [9, 10].

Proteins with GGDEF and EAL domains have been discovered as c-di-GMP receptors [11], in addition to various transcription-factor families and riboswitches located in the 5'-untranslated region of mRNA [12]. Animals, humans, and other eukaryotic systems interact with bacteria in various ways, including symbiotic and non-symbiotic associations [13]. Bacterial c-di-GMP in these hosts can also serve as a nucleotide-based second messenger for various positive or negative cellular functions [14]. Studies have shown that the host receptor interacts with c-di-GMP, activating host immune systems to defend against intracellular bacterial infections [15, 16].

Understanding how the bacterial c-di-GMP functions as a universal signaling molecule at the interspecies, intraspecies, and interkingdom levels are essential due to its critical role in regulating various cellular processes. This review discusses several types of receptors that interact with c-di-GMP to perform various biological activities at the interspecies, intraspecies, and interkingdom levels. The evolutionary relationships between these receptors have been investigated to understand their c-di-GMP specificities. The residues of receptors implicated in c-di-GMP binding are also summarized.

### Structural insight into the binding of c-di-GMP with their cognate receptors

The number of crystal structures of c-di-GMP receptors in alliance with c-di-GMP in various species is increasing [17]. Based on these structures, three types of domains containing several c-di-GMP binding motifs have been articulated [18]. Interestingly, the sorts of interactions between c-di-GMP and its receptors vary according to the type of c-di-GMP [19]. c-di-GMP interacts with its receptor as a mono, di, or tetramer. The c-di-GMP structure can also achieve a stacked or extended form as a result of variations in their torsional or glycosidic angles. In c-di-GMP receptor dimers, guanine bases may be entirely or partially stacked or de-stacked, enabling them to interact with the hydrophobic amino acid residues of a receptor. Various non-covalent interactions between c-di-GMP and its diverse receptors include hydrogen bonds, π–π, polar–π, hydrophobic–π, cation-π, anion–π, C-H bond–π, and lone pair–π interactions [17]. Each guanine base in c-di-GMP has Watson–Crick and Hoogsten edges that bind to glutamate or aspartate and arginine, respectively [20]. Specifically, the arginine of the receptor forms a hydrogen bond with the N7 and O6 atoms of the guanine Hoogsteen edge. Contrastingly, guanine's N1 and N2 atoms at the Watson–Crick edge bind to aspartic acid and glutamate. Examples of these interactions are presented below.

**Fig. 1:**
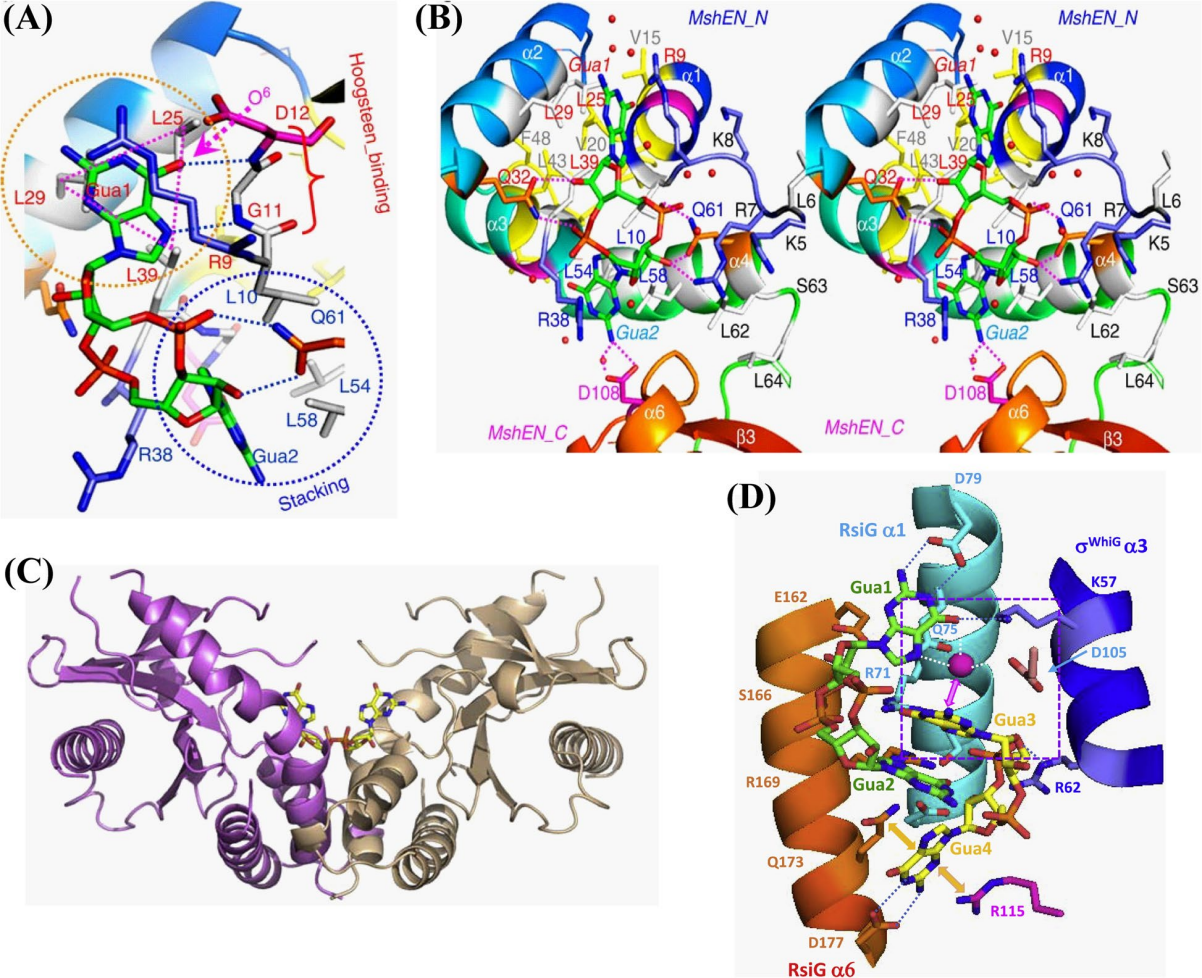
Hydrophobic interaction between c-di-GMP and MshEN receptor (**A** and **B**). Reproduced with permission from reference [21]. Copyright, 2016, Springer Nature, (**C**) Interaction of c-di-GMP with the dimer of STING in the symmetry. Reproduced with permission from reference [23]. Copyright, 2012, Elsevier Inc. and Copyright, and (**D**) The multiple non-covalent interactions of c-di-GMP dimer with the σ^WhiG^ and two α helices of RsiG in *Streptomyces venezuelae*. Reproduced with permission from reference [20]. Copyright, 2020, Elsevier Inc

The interaction between c-di-GMP and the MshEN receptor of *Vibrio cholerae* is an example of a hydrophobic interaction [21]. MshEN contains a 53-residue long domain consisting of two 24-residue motifs [RLGXX(L)(V/I)XXG(I/F)(L/V)XXXXLXXXLXXQ] connected by a 5-residue spacer that accommodates c-di-GMP. The six well-conserved residues (in bold) play an important role in the c-di-GMP binding [21]. The arginine (R9) in RLG interacts with Gua1 of c-di-GMP through stacking, whereas the leucine forms a hydrophobic triangular cluster with the L54 and L58 in the second motif. This cluster also involves hydrophobic CH-π interactions with Gua2 that stabilize protein–ligand interactions [21, 22]. Glycine residues from the conserved motif and the remaining non-conserved residues establish two hydrogen bonds with N7 and O6 of Gua1 at the Hoogsteen edge (Fig. 1A). The four-helix MshEN_N also forms a bundle structure maintained by hydrophobic residues in its core (Fig. 1B).

The stimulator of the interferon genes (STING) protein found in animals, mammals, and humans provides an example of arginine-guanine cation-π and guanine-guanine π-π interactions between c-di-GMP and its receptor [23]. A single c-di-GMP molecule is symmetrically linked to the STING dimer, with each stack formed by two guanines (Fig. 1C). STING dimers contain a c-di-GMP binding pocket formed by α1, α3, and the loop formed by β2 and β3 (Fig. 1C). The four-layer stack is formed by the stack of the guanidinium group of arginine with the guanine base and the interaction of the long side chain of arginine with Y167 [20]. It has been discovered that interactions between c-di-GMP and many residues, including P264, T263, E260, Y163, and S162, entail several Van der Waals interactions and hydrogen bonding [23]. This four-layer stack consists of Tyr/Gua/Arg/Tyr interactions. Additionally, because the guanine bases are partly stacked, a compact core ring is adopted when c-di-GMP is connected to the STING interface.

**Fig. 2:**
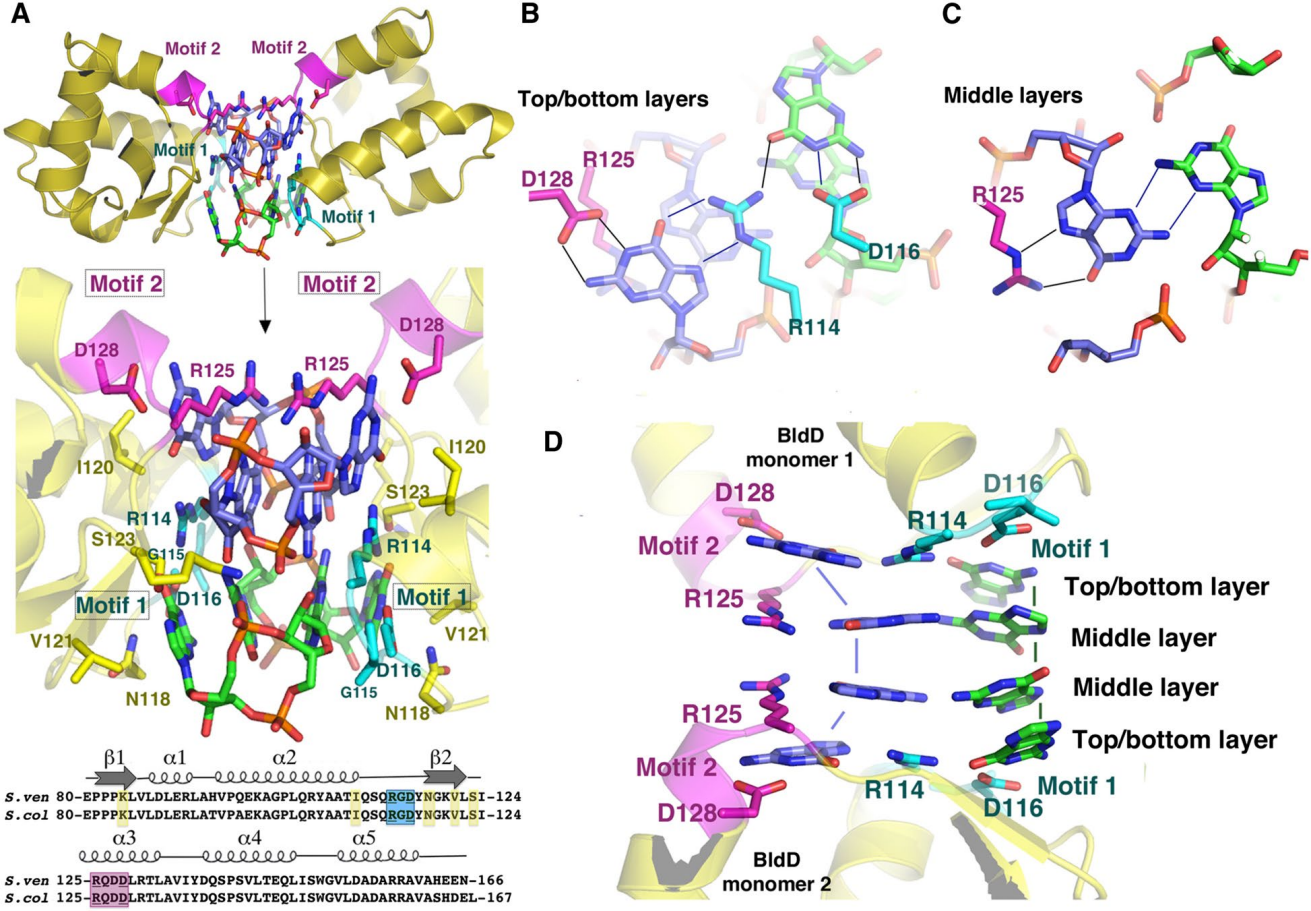
Interaction of tetrameric c-di-GMP with the two subunits of BldD via C-terminal domain with the involvement of bipartite RXD-X_8_-RXXD signature. (**A**) Structural details of BldD dimer-(c-di-GMP) complex, (**B**) Top and bottom layer of BldD C-terminal domain-(c-di-GMP) contact, (**C**) Middle layers of two c-di-GMP dimers that are intercalated, and (**D**) c-di-GMP layers in side view. Reprinted with permission from reference [25]. Copyright, 2014, Elsevier Inc

The interaction between c-di-GMP and the anti-sigma factor RsiG in combination with the sigma factor σ^WhiG^ at its interface involves lone pair–π interactions, polar–π interactions, and hydrophobic–π interactions [20]. These interactions have been reported in *Streptomyces venezuelae*, where it sequesters sporulation σ^WhiG^, inhibiting the transcription of late sporulation genes [24]. Figure 1D shows interactions between the c-di-GMP dimer and σ^WhiG^, in addition to two EXXXSXXRXXXQXXXD motifs from the α1 and α6 helices of RsiG. The two antiparallel helices of RsiG are aligned together, and residues such as glutamic acid, arginine, and aspartic acid present in the EXXXSXXRXXXQXXXD motif establish hydrogen bonds with the two c-di-GMP monomers. The two extended guanines and the Gln residues within this motif have been seen to interact non-covalently. Additionally, a water molecule also helps to stabilize the c-di-GMP dimer by creating two hydrogen bonds with Gua1's N7 and O6 atoms in each c-di-GMP monomer, one with the carboxyl oxygen of adjacent D105 and the other with Gua3 in the second c-di-GMP monomer. *S. venezuelae* BldD is another receptor that interacts with c-di-GMP through π–π interaction and helps to transition hypha-spore forms by suppressing BldD regulon genes during vegetative growth [25] (Fig. 2).

### Biological roles of c-di-GMP receptors in prokaryotic systems

**Fig. 3:**
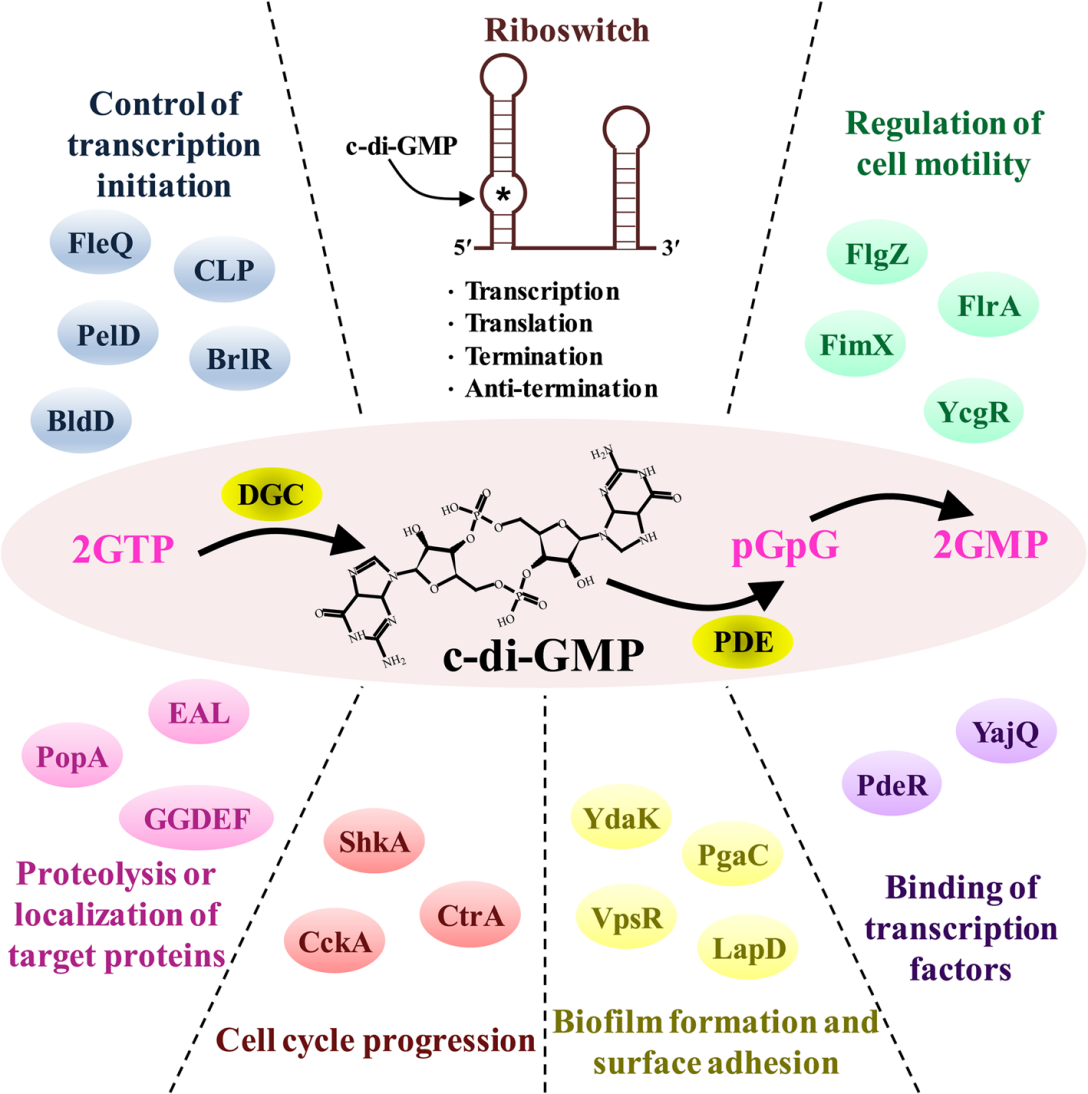
Interaction of c-di-GMP with different receptor proteins for controlling diverse biological roles in the bacterial system. The information obtained from the literature [28–30]

In the bacterial system, c-di-GMP helps with the transduction of external (e.g., temperature, energy, surfaces, light, redox potential, respiratory electron acceptors, and several natural and synthetic chemicals) [26] or internal (generated during developmental progression or cellular division) [27] signals by binding to the effector component or receptor. These c-di-GMP-bound receptors/effectors specifically interact with their targets, triggering a wide range of cellular processes. Moreover, c-di-GMP has demonstrated various biological functions, including motility, the formation and dispersion of biofilm, cell division, differentiation, quorum sensing, and virulence [4]. Here, we discuss the functional consequences of the interaction of c-di-GMP with its effectors or receptors (Fig. 3). Table 1 summarizes the types of c-di-GMP receptors found in prokaryotic and eukaryotic systems and the outcome of their interactions in terms of biological function. Table 1 also highlights the binding domains or motifs associated with interaction of c-di-GMP with its receptors. The outcome of this interaction is classified into the categories of control of transcription initiation and termination, control of translation, control of exopolysaccharide (EPS) synthesis and secretion, control of flagellar and type III secretion system assembly, control of cell cycle progression, proteolysis of target proteins, and involvement in the localization of target proteins [28]. Detailed descriptions of the biological functions of these outcome reactions are provided below.

**Table 1:** Different types of c-di-GMP receptors from prokaryotes and eukaryotes

Name of receptor	Sources	Binding domain	Binding affinity	Functional properties	References
**Bacteria**					
Tlp1	*Azospirillum brasilense*	PilZ domain	**-**	Promoted persistent motility	[31]
Cbp1	*A. caulinodans*	PilZ domain	14.94 μM	Regulated motility, biofilm formation, and virulence	[9]
YkuI	*Bacillus subtilis*	EAL domain	**-**	**-**	[32]
YdaK	*B. subtilis*	GGDEF domain	1.1 µM	**-**	[33]
YdaK	*B. velezensis*	GGDEF domain	**-**	Regulated biofilm formation and bacterial colonization	[34]
PlzA	*Borrelia burgdorferi*	PilZ domain	6.25 μM	Required to survived within ticks	[35]
PlzB	*B. burgdorferi*	PilZ domain	0.002 μM	Enhanced biological fitness	[36]
PlzA	*B. burgdorferi*	PilZ domain	**-**	Multiple cellular activities were regulated, including osmolality sensing, motility, and nutrition usage	[37]
PlzA	• *B. burgdorferi* • *B. hermsii*	xPliZ domain	6.25 μM	Carried out environment-specific roles in *Borreliella* biology	[38]
Bcam1349	*Burkholderia cenocepacia*	GGDEF and EAL domain	10 μM	• Regulated production of cellulose and fimbriae • Regulated biofilm formation and virulence	[39]
RpfR	*B. cenocepacia*	EAL domain	2.92 μM	**-**	[40]
YajQ	*B. gladioli*	**-**	**-**	Mediated endophytic mobility-based defense for host	[41]
PleD	*Caulobacter crescentus*	DGC domain	**-**	**-**	[42]
DgrA	• *C. crescentus* • *Salmonella* Typhimurium • *Pseudomonas aeruginosa*	PilZ domain	< 0.05 μM	Regulated cell motility control	[43]
CbrR	*Campylobacter jejuni*	**-**	**-**	Acted as a virulence factor in the pathogenesis	[44]
PopA	*C. crescentus*	GGDEF domain	2 μM	Controlled the cell cycle	[45]
ShkA	*C. crescentus*	Pseudo-receiver domain	**-**	• TacA transcription factor activation • Started a G1/S-specific transcription program, resulting in cell morphogenesis and S-phase entry	[46]
TipF	*C. crescentus*	EAL domain	32.5 μM	Formed polar flagellar assembly	[47]
CckA	*C. crescentus*	**-**	4.7 μM	• Inhibited kinase activity • Stimulated phosphatase activity	[48]
Riboswitch class-II	*Clostridium difficile*	**-**	**-**	Involved in the ribozyme self-splicing	[49]
PilB2	*C. perfringens*	**-**	1.34 μM	**-**	[50]
BcsA	*Dickeya oryzae*	PilZ domain	0.98 μM	Controlled bacterial biofilm formation	[51]
YcgR	*D. oryzae*	PilZ domain	-	Modulated the bacterial motility phenotype by increasing putrescine levels	[52]
BcsA	*Erwinia amylovora*	PilZ domain	-	Activated cellulose biosynthesis	[53]
CsrD	*E. amylovora*	EAL domain	1.7 μM	Contributed to virulence and biofilm formation	[54]
PNPase	*Escherichia coli*	**-**	2.9 μM	Catalyzed phosphorolysis of RNA	[55, 56]
• PgaC • PgaD	*E. coli*	**-**	0.062 μM	Induced biofilm formation	[57]
BcsE	*E. coli*	GIL and GGDEF I-site-like domain	2.4 μM	Required for maximal cellulose production	[58]
BolA	*E. coli*	**-**	**-**	• Enhanced survivability under various circumstances • Involved in the production of biofilms	[59]
MrkH	*Klebsiella pneumoniae*	PilZ and MrkH N domain	0.107 μM	Promoted biofilm formation	[60]
• WspR • RpfG	*Lysobacter enzymogenes*	REC domain joined to GGDEF and *N*-terminal REC domain linked to C-terminal HD-GYP domain	0.15 μM	Regulated biofilm formation	[61]
ArgR	*Mycobacterium bovis*	**-**	0.34 μM	Triggered *Mycobacterium* to adapt to hypoxia through	[62]
LtmA (MSMEG-6479)	*M. smegmatis*	TetR-type HTH domain	0.83 μM	Regulated cell wall permeability and cell wall composition	[63]
DarR (MSMEG-5346)	*M. smegmatis*	HTH DNA-binding domain at the N-terminus, QacR-like domain at the C-terminus	2.3 μM	**-**	[64]
HpoR (MSMEG_5860)	*M. smegmatis*	**-**	1.78 μM	Enhanced the mycobacterial H_2_O_2_ resistance	[65]
• LtmA (MSMEG-6479) • HpoR (MSMEG_5860)	*M. smegmatis*	**-**	• 0.62 μM for LtmA • 0.29 μM for HpoR	Enhanced bacterial growth under antibiotic-stressful conditions	[66]
DevR	*M. smegmatis*	C-terminal DNA-binding domain	1.96 μm	Involved in the regulation of mycobacterial oxidative adaptation	[67]
PdtaS	*M. tuberculosis*	GAF domain	0.33 μM	**-**	[68]
SgmT	*Myxococcus xanthus*	GGDEF domain	**-**	**-**	[69]
CdbA (MXAN_4361)	*M. xanthus*	RHH DNA binding domain	~ 0.083 μM	Contributed to chromosome organization	[70]
CdbS	*M. xanthus*	PilZ domain	~ 1.4 μM	Heat stress accelerates chromosomal disorganization and cell death	[71]
PilZ	*P. aeruginosa*	PilZ domain	**-**	• Produced functional pili • Biosynthesized exopolysaccharide • Regulated flagellar motor activity • Expressed virulence gene	[72–74]
Alg44	*P. aeruginosa*	PilZ domain	**-**	Biosynthesized alginate	[75–78]
WspR	*P. aeruginosa*	GGDEF domain	**-**	Controlled biofilm formation	[79, 80]
FimX	• *P. aeruginosa* • *Xanthomonas citri* • *X. oryzae* pv.* oryzae*	GGDEF and EAL domain	0.1–0.2 μM	Regulated twitching motility, biofilm formation, and bacterial virulence expression	[18, 81–84]
PelD	*P. aeruginosa*	GAF and GGDEF domain	0.5–1.9 μM	Produced Pel polysaccharide	[76, 85]
FleQ	*P. aeruginosa*	*N*-terminal FleQ, central AAA + ATPase, and C-terminal HTH DNA-binding domain	7 μM	Down-regulated flagella gene expression	[86]
BrlR	*P. aeruginosa*	HTH_BrmR and Gyrl-like domain	2.2 μM	Contributed to the high-level drug tolerance of biofilms	[87]
FimX	*P. aeruginosa*	EAL domain	0.09 μM	Type IV pili assembly and twitching motility are regulated	[88]
FlgZ	*P. aeruginosa*	PilZ domain	**-**	Controlled swarming motility	[89]
BrlR	*P. aeruginosa*	DNA-binding domain	7.3 μM	Mediated antibiotic resistance	[90]
MapZ	*P. aeruginosa*	PilZ domain	μM	• The flagellar motor switching frequency was reduced • Surface attachment occurs during biofilm development	[91]
HapZ	*P. aeruginosa*	PilZ domain	2.0 μM	Mediated bacterial motility and biofilm formation	[10]
LapD	*P. fluorescens*	EAL domain	5.5–13.1 μM	Required for biofilm development and stable surface adhesion	[92–94]
• LapD • LapG	*P. fluorescens*	EAL domain	1.9 µM	Controlled cell adhesion and biofilm formation	[95]
YcgR	• *S.* Typhimurium • *E. coli*	PilZ domain	0.18–0.84 μM	Regulated flagellum-based motility	[8, 19, 43, 96]
BcsA	• *S.* Typhimurium • *E. coli*	PilZ domain	8.2 μM	Stimulated bacterial cellulose production	[96, 97]
BgsA	*Sinorhizobium meliloti*	C-terminal cytoplasmic domain	**-**	Encoded glycosyl transferase	[98]
CuxR	*S. meliloti*	Cupin domain	6.7 µM	Induced expression of EPS biosynthesis gene cluster	[99]
SaCpaA_RCK	*Staphylococcus aureus*	RCK domain	9 μM	Regulated ion transport	[100]
KdpD	*S. aureus*	USP domain	2 μM	Involved in the control of the two primary K^+^ uptake systems' activity and expression	[101]
FsnR (Smlt2299)	*Stenotrophomonas maltophilia*	REC and HTH domain	3.43 μM	Elicited flagellar gene expression	[102]
BldD	• *Streptomyces coelicolor* • *S. venezuelae*	**-**	2.5 μM	Repressed expression of sporulation genes	[25]
BldD	*S. ghanaensis*	**-**	**-**	Played a role in morphological differentiation	[103]
GlgX	*S. venezuelae*	C-domain	~ 8 µM	Stimulated the enzyme's catalytic activity to hydrolyze glycogen	[104]
TDE0214	*Treponema denticola*	PilZ domain	1.73 μM	Played roles in spirochete motility, biofilm formation, and pathogenicity	[105]
• PlzC (VC_2344) • PlzD (VCA0042)	*Vibrio cholerae*	PilZ domain	0.1–0.3 μM	Regulated biofilm formation, motility, and virulence	[106, 107]
VpsT	*V. cholerae*	REC and HTH domain	3.2 μM	• Promoted biofilm formation • Down-regulated motility genes	[108]
VpsR	*V. cholerae*	ATP binding and HTH DNA binding domain	1.6 μM	Controlled biofilm development	[109]
FlrA	*V. cholerae*	*N*-terminal receiver, AAA + , and C-terminal DNA-binding domain	0.38 μM	Regulated flagellar biosynthesis	[110]
MshEN	*V. cholerae*	MshE *N*-terminal domain	0.014–2 µM	Direct contact with related type II secretion and type IV pili ATPases regulates membrane complexes	[21, 111]
GbpA	*V. cholerae*	**-**	**-**	Attached to environmental and host surfaces containing *N*-acetylglucosamine moieties	[112]
TfoY	*V. cholerae*	TfoX *N*-terminal domain	**-**	Regulated natural competency, type VI secretion system, and motility	[113]
Riboswitch class-I	*V. cholerae*	**-**	**-**	Helped in RNA compaction and structural rearrangement	[114]
BrpT	*V. vulnificus*	**-**	135.4 μM	Enhanced cell-surface adherence	[115]
CLP	*X. campestris*	**-**	3.5 μM	Regulated bacterial virulence gene expression	[116]
XC_3703	*X. campestris* pv. *campestris*	**-**	2 µM	Activated virulence-related genes	[117]
• PXO_00049 • PXO_02374 • PXO_02715	*X. oryzae* pv. *oryzae*	PilZ domain	• 0.139 μM for PXO_00049 • 0.102 μM for PXO_02374	Regulated virulence	[118]
PilZX3 (PXO_02715)	*X. oryzae* pv. *oryzae*	GGDEF amd EAL domain	**-**	Regulated virulence	[119]
GdpX6 (PXO_02019)	*X. oryzae* pv. *oryzae*	GGDEF domain	9 μM	Controlled the virulence, swimming and sliding motility, and biofilm formation	[120]
**Cyanobacteria**					
CdgR	*Anabaena* PCC 7120	**-**	**-**	Regulated cell size	[121]
Cellulose synthase Tll0007	*Thermosynechococcus vulcanus*	PilZ domain	63.9 µM	Being necessary for cell aggregation	[122]
**Plants**					
atCNTE1	• *Arabidopsis thaliana* • *Oryza sativa*	CNB and GAF domain	**-**	**-**	[123]
Cellulose synthase	*Gossypium hirsutum*	**-**	13–24 μM	Involved in cellulose synthesis	[124]
**Animal**					
LcSTING2	*Larimichthys crocea*	TMEM173 domain	**-**	Exhibited immune response against parasites	[125]
MPYS	Mouse	**-**	**-**	Induced cytokine expression	[126]
**Other eukaryotes**					
P21^ras^	Jurkat cells	**-**	**-**	Expressed the CD4 receptor	[127]
STING	HEK293T cells	Amino-terminal domain	~ 5.65 μM	• Functioned as immunosensor • Elicited IFN production	[23, 128, 129]
Coronin 1A	Mammalian macrophages	Nucleotide-binding domain	**-**	• Induced IFN-I expression • Stimulated inflammatory responses	[130]
Cyclophilin H	Mammalian macrophages	Nucleotide-binding domain	**-**	• Induced IFN-I expression • Stimulated inflammatory responses	[130]
Heliase DDX41	Mouse and human cells	DEAD-box domain	~ 5.65 μM	STING triggered the type I IFN host immunological response	[131]
STING	*Drosophila melanogaster*	C-terminal domain	**-**	Initiated innate immune response	[132]

#### Biofilm formation and bacterial colonization

Bacteria use c-di-GMP signaling to transition from a motile to a solitary existence in response to environmental cues [133]. Bacterial receptor proteins associate with c-di-GMP to create the extracellular cellulose matrix of biofilms [134]. For instance, Bcam1349 protein from *Burkholderia cenocepacia*, combined with c-di-GMP, promotes pellicle and biofilm architecture progression [39]. It has been shown that c-di-GMP increases the Bcam1349 protein's affinity for the promoter of a target gene, which results in the development of biofilms. In *Pseudomonas aeruginosa*, the binding of MapZ to c-di-GMP alters motility by controlling the switching frequency [91]. This interaction is also related to enhanced adhesion during biofilm formation. Additionally, HapZ interacts with SagS in *P. aeruginosa* in a c-di-GMP-dependent way to promote surface adhesion and biofilm formation [10]. This is consistent with the role of SagS in controlling surface adhesion during the early stages of biofilm formation. In *Escherichia coli*, c-di-GMP stimulates biofilm formation by directly binding to PgaC and PgaD [57]. The Pga machinery triggers the synthesis of poly-β-1, 6-*N*-acetylglucosamine, a biofilm component of *E. coli*. c-di-GMP also stimulates the expression of the gene encoding MrkH (a key factor in biofilm formation) in *Klebsiella pneumoniae* [60]. Here, the C-terminal- and *N*-terminal domains of MrkH promote biofilm development upon the mediation of dimer association by c-di-GMP. Similarly, *V. cholerae* biofilm development is aided by VpsT-sensing c-di-GMP via extracellular matrix synthesis regulation [108]. VpsT downregulates the motility genes of *V. cholerae* in a c-di-GMP-dependent fashion, leading to a sessile colony state. In addition, BrpT in *V. vulnificus* binds directly to c-di-GMP and promotes the expression of EPS, thereby improving cell surface adhesion [115]. BolA interacts with c-di-GMP and promotes motility and biofilm formation, allowing *E. coli* to transition between planktonic and sessile lifestyles [59]. Even in the presence of high c-di-GMP levels, the lack of BolA resulted in less robust biofilm development, emphasizing the importance of BolA in this regard. Alg44 in *P. aeruginosa* is involved in the biosynthesis of the EPS alginate via binding to c-di-GMP [75, 76, 135]. It has also been demonstrated that c-di-GMP regulates polysaccharide polymerization and transport via Alg44. In *Sinorhizobium meliloti*, CuxR stimulates EPS production at high doses of c-di-GMP [99]. TDE0214 protein produces a biofilm and exhibits virulence in the periodontal pathogen *Treponema denticola* via the c-di-GMP signaling cascade [105]. Here, TDE0214 plays a significant role in cell adhesion and colonization during the early stages of biofilm formation.

#### Bacterial survival and adaptation

Several bacterial species respond to environmental fluctuations via the c-di-GMP signaling system [136]. In addition, the presence of c-di-GMP in bacteria promotes host adaptation and persistence by regulating bacterial metabolism [137]. Tlp1 from *Azospirillum brasilense* promotes motility by binding to c-di-GMP through its C-terminal PilZ domain. This allows the microaerobic *A. brasilense* to reach a hypoxic environment [31]. ArgR from *Mycobacterium bovis* modulates c-di-GMP signaling to adapt to hypoxic environments [62]. Specifically, c-di-GMP mitigates the autorepression of ArgR expression and promotes nitrite respiration, thereby increasing the viability of *M. bovis* in hypoxic environments. c-di-GMP in *M. smegmatis* enhances their antioxidant resistance by binding to HpoR and LtmA [65, 66]. Here, c-di-GMP is upregulated by high levels of H_2_O_2_ and enhances antioxidant resistance by specifically binding to HpoR, which induces DNA-binding activity. In addition, LtmA enhances the growth of *M. smegamatis* under high antioxidant stress levels, in contrast to HpoR. *Borrelia burgdorferi*, a tick-borne pathogen, also enhances its host adaptability by binding PlzA to c-di-GMP [35]. These results are attributed to the effector mechanism that occurs through PlzA—c-di-GMP interaction [36].

#### Regulation of bacterial pathogenesis

c-di-GMP regulates factors that affect bacterial pathogenesis, including the expression of virulence genes, biofilm formation, and motility regulation [138]. The diversity of c-di-GMP receptors and effectors contributes to bacterial lifestyle transformation by regulating multiple cellular processes [139]. PlzC and PlzD in *V. cholerae* particularly cooperate with c-di-GMP to control the expression of the virulence genes and the production of biofilms [106]. PlzC and PlzD proteins regulate downstream processes by binding to c-di-GMP. Additionally, VpsR is connected to c-di-GMP and activates *aphA* and *vpsT* to govern biofilm development and virulence factor production [109]. FlrA controls the motility of *V. cholerae* by downregulating flagellar biosynthesis [110]. Furthermore, c-di-GMP inhibits *V. cholerae* motility by promoting the formation of extracellular *Vibrio* polysaccharides. WspR from *P. aeruginosa* regulates biofilm development by binding to c-di-GMP [79]. This is attributed to c-di-GMP, which restricts the mobility of the GGDEF domain of WspR. Furthermore, in the presence of c-di-GMP, *P. aeruginosa* reduces the expression of flagellar genes [86]. This effect is enhanced in the presence of FleN. Finally, *E. coli* YcgR also binds to c-di-GMP to regulate flagella-based motility [8].

#### Regulation of enzyme activity

c-di-GMP may also bind to enzymes and thus regulate their activities [27]. Cellulose production by pathogens is a key factor influencing virulence, since cellulose is a fundamental component of biofilm structures [140]. In *B. cenocepacia*, c-di-GMP controls cellulose synthesis by increasing the binding of Bcam1349 to the cellulose synthase gene [39]. BcsA controls cellulose synthesis in *Erwinia amylovora* in a c-di-GMP-dependent fashion [53]. Here, the allosteric binding of c-di-GMP to BcsA contributes to biofilm formation by activating cellulose biosynthesis. BcsE from *E. coli* maximizes cellulose production by binding to c-di-GMP through its DUF2819 domain [58]. Additionally, BcsA and BcsB form a complex to activate c-di-GMP, thereby upregulating cellulose synthesis in *E. coli* [97]. This occurs by activating the auto-inhibited enzyme by breaking the salt bridges that bind the gating loops controlling access to the active site and substrate coordination. Finally, GlgX from *S. venezuelae* interacts with c-di-GMP to stimulate the enzymatic hydrolyzation of glycogen [104]. This indicates that the GlgX-c-di-GMP interaction is vital for controlling glucose availability in *Streptomyces*.

### Biological roles of c-di-GMP receptors in eukaryotic systems

**Fig. 4:**
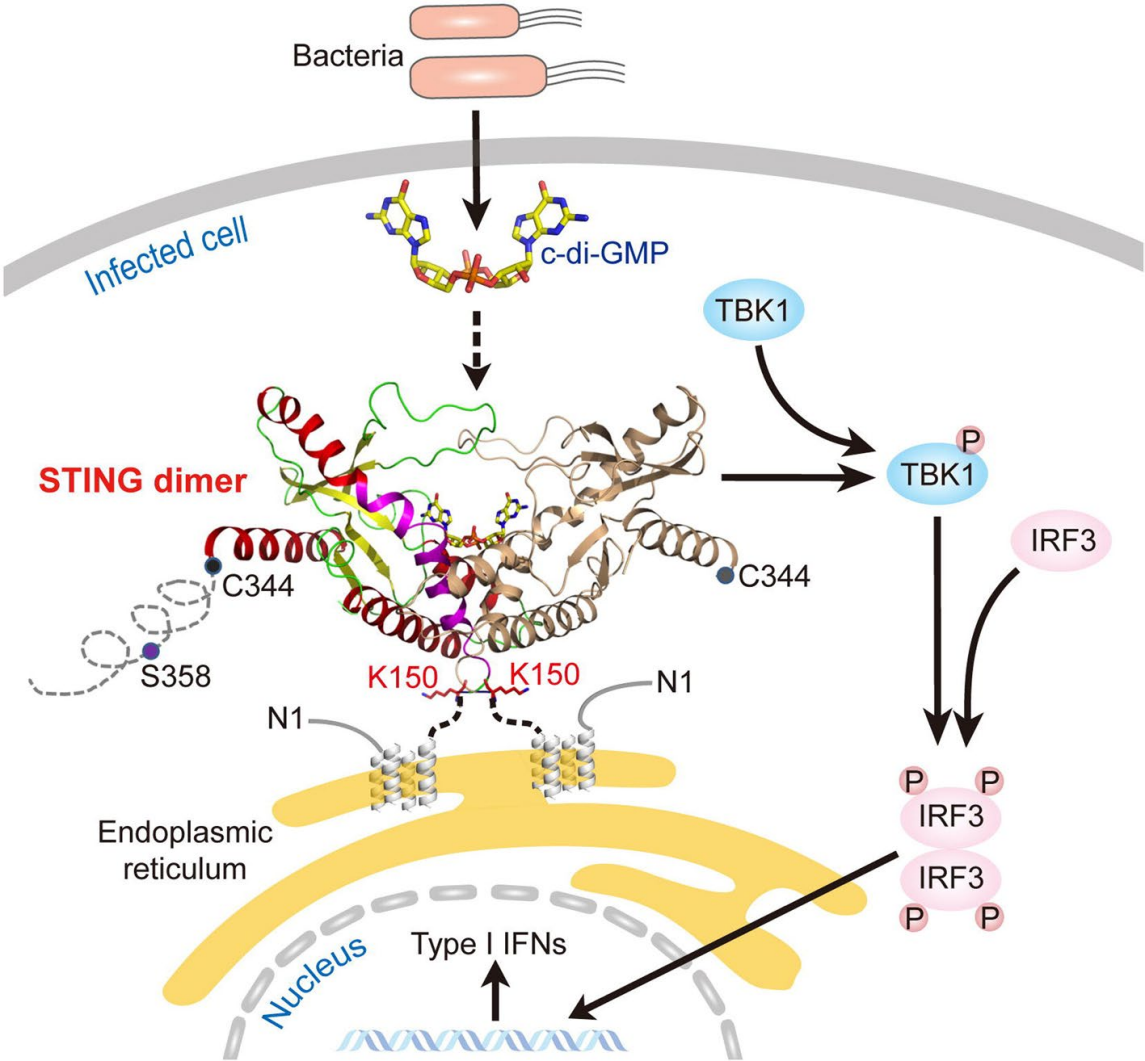
Downstream signaling induced by bacterial c-di-GMP binding to STING cognate receptors leads to the generation of type I IFNs through the IFN regulatory factor 3 and TANK-binding kinase 1 pathway. Reprinted with permission from the reference [129]. Copyright, 2012 Elsevier Inc

Bacterial c-di-GMP can interact with eukaryotic cell systems as well [104]. c-di-GMP is used by eukaryotes as a pathogen presence indicator [27]. In this regard, c-di-GMP modulates the response of the eukaryotic immune system to microbial pathogens [14]. In mammals, STING acts as an immune sensor by inducing a type I interferon (IFN) response by binding to c-di-GMP [23, 128]. Furthermore, binding to c-di-GMP improves the interaction between the C-terminal domain of STING and TANK-binding kinase 1, resulting in IFN production [129] (Fig. 4). DDX41 (a c-di-GMP-detection pattern recognition receptor) encourages connections with STING, enhances STING's affinity for c-di-GMP, and activates IFNs [131]. STING from *Larimichthys crocea* (a large yellow croaker) enhances its immunity against the infection caused by the parasite *Cryptocaryon irritans,* by acting as a receptor for c-di-GMP [125]. During *C. irritans* infection, STING mediates the production of type I IFNs and inflammatory cytokines. In *Itgax*^Cre^*Tmem173*^Flox/Flox^ mice, c-di-GMP induces cytokine production by selectively activating cells expressing the mammalian c-di-GMP receptor MPYS (STING, MITA, and TMEM173) [126]. In Jurkat cells, c-di-GMP binds to the P21^ras^ protein and stimulates CD4 receptor expression [127]. Specifically, c-di-GMP binding to P21^ras^ maintains the protein in an active form following CD4 overexpression. In mammalian macrophages, coronin 1A and cyclophilin H bind to c-di-GMP and induce type I IFN expression, activating an inflammatory response [130]. These findings suggest that mammalian macrophages detect and respond to c-di-GMP-induced inflammation. Finally, the membrane polypeptide of cellulose synthase from cotton fibers (*Gossypium hirsutum*) was characterized using the same c-di-GMP receptor as the cellulose synthase from *Acetobacter xylinum* [124]. Plant cellulose synthase membrane polypeptides are identical to bacterial cellulose synthase subunits, allowing for the identification of genes responsible for cellulose synthesis in plants.

### Evolutionary relationships between bacterial c-di-GMP receptors and identification of the bidirectional best hit

**Fig. 5:**
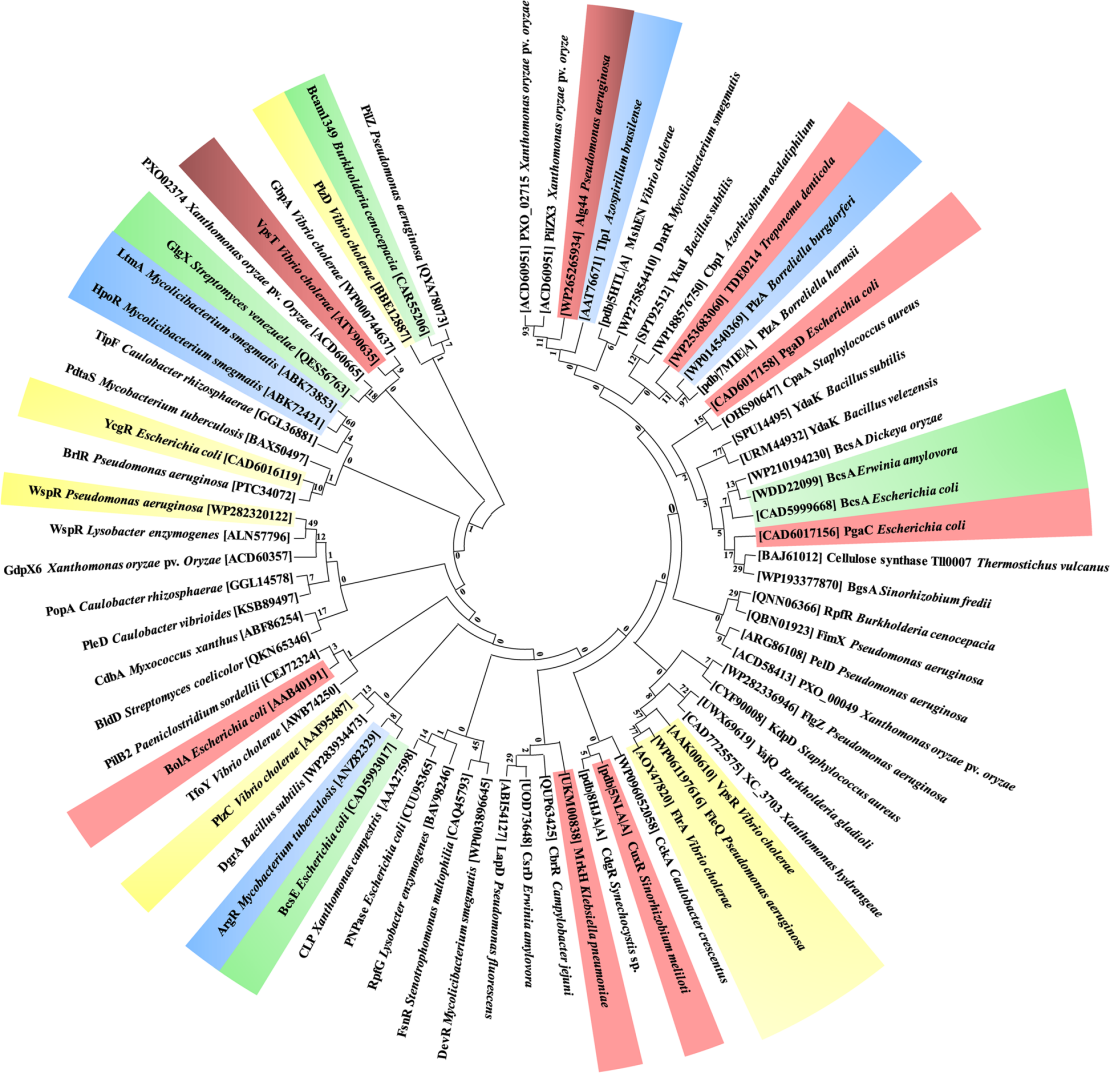
Phylogenetic tree of the c-di-GMP receptors from different species of bacteria. The red-colored proteins promote biofilm formation, the blue-colored proteins promote bacterial survival and adaptability, the yellow-colored proteins promote bacterial pathogenicity, and the green-colored proteins promote enzyme activity

To investigate the evolutionary relationship of c-di-GMP receptors, a phylogenetic tree of receptors from several bacterial species was constructed (Fig. 5). Subsequently, the best bidirectional hit among these receptors was determined to demonstrate how these receptors from various bacterial species are clustered in the same branch of the tree. The bidirectional best hit helps to determine how the proteins share similar affinities for the same chemical (c-di-GMP). YcgR is recognized as a flagellar brake protein due to its role in controlling swarming and swimming in various flagellated bacterial species by interacting with c-di-GMP [141, 142]. YcgR from *E. coli* was found to be clustered with BrlR from *P. aeruginosa* in the same branch in the phylogenetic tree developed. In *P. aeruginosa*, BrlR regulates biofilm formation and multidrug efflux pump production, in addition to acting as a pyocyanin receptor [90]. However, a sequence similarity analysis revealed no similarities between the two proteins. Further, YcgR from *E. coli* was found to show 82.35% similarity to FlgZ from *P. aeruginosa*, indicating potentially similar binding specificities for c-di-GMPs and a role in flagellar motility (Table 2).

**Table 2:** Bidirectional best hit of among c-di-GMP receptors from different bacterial species

Bacterial species	Protein	Bacterial species	Protein	% Of identity
*Azospirillum brasilense*	Tlp1	*Bacillus subtilis*	YkuI	100.00
*B. subtilis*	YdaK	*B. velezensis*	YdaK	87.94
*Burkholderia cenocepacia*	RpfR	*Caulobacter vibrioides*	CckA	53.33
*C. vibrioides*	CckA	*Campylobacter jejuni*	CbrR	71.43
*Dickeya oryzae*	BcsA	*Erwinia amylovora*	BcsA	91.95
*C. jejuni*	CbrR	*E. amylovora*	CsrD	57.14
*E. amylovora*	CsrD	*Escherichia coli*	PgaC	66.67
*E. coli*	PgaC	*E. coli*	PgaD	57.14
*E. coli*	BolA	*Klebsiella pneumoniae*	MrkH	66.67
*K. pneumoniae*	MrkH	*Mycobacterium tuberculosis*	PdtaS	50.00
*Lysobacter enzymogenes*	WspR	*Pseudomonas aeruginosa*	WspR	72.22
*P. aeruginosa*	WspR	*P. aeruginosa*	FleQ	54.55
*P. aeruginosa*	FlgZ	*E. coli*	YcgR	82.35
*E. coli*	YcgR	*Staphylococcus aureus*	KdpD	55.56
*Streptomyces coelicolor*	BldD	*S. venezuelae*	GlgX	100.00
*S. venezuelae*	GlgX	*Treponema denticola*	TDE0214	55.56
*T. denticola*	TDE0214	*Vibrio cholerae*	VpsR	50.00
*Pseudomonas aeruginosa*	FleQ	*V. cholerae*	FlrA	61.54
*V. cholerae*	VpsR	*V. cholerae*	GbpA	66.67
*V. cholerae*	GbpA	*Xanthomonas oryzae* pv. *oryzae*	PXO00049	50.00
*B. cenocepacia*	Bcam1349	*C. rhizosphaerae*	PleD	71.43
*C. rhizosphaerae*	PleD	*Paeniclostridium sordellii*	PilB2	56.25
*D. oryzae*	BcsA	*E. coli*	BcsA	69.68
*D. oryzae*	BcsA	*Sinorhizobium fredii*	BcsA	52.73
*E. amylovora*	BcsA	*E. coli*	BcsA	69.24
*E. amylovora*	BcsA	*S. fredii*	BcsA	52.22
*B. gladioli*	YajQ	*X. hydrangeae*	XC3703	65.84

PlzA is a protein that binds to c-di-GMP (29.6 kDa, 261 amino acids) found in many species of Lyme disease spirochetes, such as *Borreliella* spp. Multiple mechanisms were hypothesized to influence borrelial activity previously. PlzAs from all *Borreliella* species were grouped together in the same branch. Moreover, LapD is a c-di-GMP-binding receptor that regulates cell surface attachment and biofilm formation [92]. LapD from *P. fluorescence* was grouped in the same branch as CsrD from *E. amylovora*, CbrR from *Campylobacter jejuni*, and MrkH from *K. pneumoniae*. However, the sequence similarities indicated that CbrR and CsrD have 57.14% similarity, whereas MrkH has 66.67% similarity with BolA from *E. coli* and 50.00% similarity with PdtaS from *M. tuberculosis*.

Various proteins help regulate biofilm development by producing extracellular polymers such as alginate and cellulose [140]. Several bacterial polymer-synthesizing proteins, such as cellulose and alginate synthases, were grouped in the same branch as well (Fig. 5). BcsA from *Dickeya oryzae*, which is involved in cellulose synthesis, showed 91.95% similarity with BcsA from *E. amylovora* (Table 2), yet lower similarities were exhibited with BcsA from *E. coli* (69.68%) and *S. fredii* (52.73%). The YdaK protein from *Bacillus subtilis*, which is involved in the production of putative EPS facilitating biofilm formation, was grouped in the same branch as that of the cellulose-producing proteins (Fig. 5). Additionally, YdaK from *B. subtilis* showed high similarity (87.94%) with the YdaK protein from *B. velezensis* (Fig. 5). Tlp1 from *A. brasilense* exhibits sustained motility after interacting with c-di-GMP via the PilZ domain and was found to be clustered along YkuI from *B. subtilis.*

Although the precise role of Ykul is unknown [143], a high degree of similarity (100.00%) between Yku1 and Tlp1 from *A. brasilense* suggests that it may also influence motility. WspR from *P. aeruginosa*, which is involved in biofilm production, was grouped in the same branch as the WspR from *Lysobacter enzymogenes* with a sequence similarity of 72.22%. BalD (controls the expression of sporulation genes in *S. coelicolor*) is found in the same branch as CdbA, which is required for viability in *Myxococcus xanthus* and plays a role in its chromosomal organization and segregation [70]. Furthermore, a sequence similarity study showed 100.00% similarity between BalD and GlgX of *S. venezuelae*, which functions as a glycogen-debranching enzyme [104]. GlgX is thought to be important in sporulation since a mutation in the *glgX* gene results in a strain that produces less spores than the wild-type strain [104]. In the phylogenetic tree, GlgX from *S. venezuelae* was grouped in the same branch as PXO02374 from *Xanthomonas oryzae* and VpsT and GbpA from *V. choloreae*. CckA (cell cycle kinase) from *Caulobacter vibrioides* [48] is found in the same cluster as CdgR (controls cell size) from *Synechocystis* sp. and CuxR (regulates EPS synthesis) from *S. meliloti* [99]. Surprisingly, CckA has a strong resemblance (71.43%) to CbrR (which regulates flagellar motility) in *C. jejuni* [44].

### Evolutionary relationship between eukaryotic c-di-GMP receptors and identification of the bidirectional best hit

**Fig. 6:**
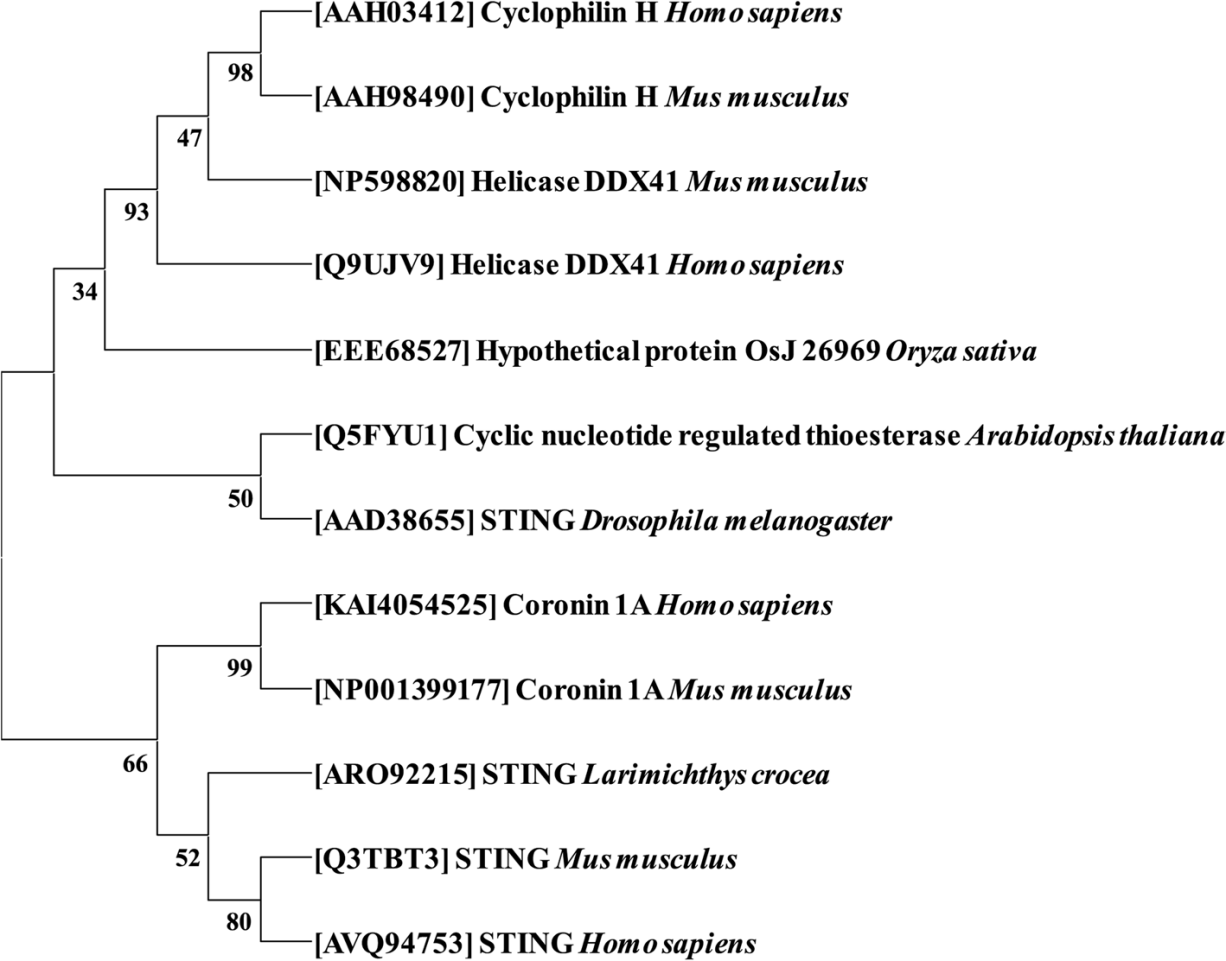
Phylogenetic tree of the c-di-GMP receptors from different eukaryotic organisms including humans

A phylogenetic tree of receptors of eukaryotic origin was also constructed (Fig. 6). The results revealed that STING from *Drosophila melanogaster* shares a high degree of similarity with STING from *Mus musculus* (100.00%), *Homo sapiens* (100.00%), and *L. crocea* (60.00%). In contrast, STING from *M. musculus* had a similarity of 88.95% with STING from *H. sapiens*. Protein helicase DDX41 from *H. sapiens* shares a high degree of similarity with *H. sapiens* coronin 1A (91.13%) and helicase from *M. musculus* (99.52%). The cyclophilin H proteins from *H. sapiens* and *M. musculus* were found in the same branch and showed 100.00% similarity.

### Methodology: phylogenetic analysis and bidirectional best hit analysis

The sequences of all receptors were retrieved from the National Center for Biotechnology Information database. After multiple sequence alignments of c-di-GMP receptors, the phylogenetic trees were constructed using MEGA version 11 software [144]. The Geneious® 11.0.2 program was used to perform the bidirectional best hit among c-di-GMP receptors and multiple sequence alignment using the BLOSUM-65 matrix.

## Conclusion and future perspectives

With recent advances in bioinformatics, structural biology, molecular biology, and biochemistry, the discovery of c-di-GMP receptors has expanded from prokaryotic to eukaryotic systems. Bacterial species use c-di-GMP signaling molecules in their natural environment through interactions with receptors, which are either membrane-bound or cytoplasmic. Bacteria are ubiquitous and can have either beneficial or detrimental effects on host systems. c-di-GMP plays a crucial role in maintaining homeostasis in the cell life cycle. Eukaryotic systems also use c-di-GMP released by bacteria to control cellular processes. Control of intracellular processes at the post-translational, translational, and transcriptional stages is a significant ability of c-di-GMP receptors. Multiple non-covalent bonding interactions influence the interaction between c-di-GMP and its receptors in many biological systems. c-di-GMP receptors bind c-di-GMP via unique domains and motifs. Here, a phylogenetic tree of c-di-GMP receptors from bacterial and eukaryotic systems was created to understand distinct receptors that participate in diverse biological processes with similar c-di-GMP binding specificities. Several common receptors found in multiple bacterial species were found to be grouped in the same branch. A significant level of sequence similarity between the receptors in various bacterial genera implies shared specificities for c-di-GMP. The functionality of various bacterial species influenced upon binding receptor proteins with c-di-GMP is also clarified in this review. The following are the key aspects that must be addressed in future studies to better understand the mechanism of interaction between c-di-GMP and its receptors, as well as their diverse functional characteristics.

- The receptor in pathogenic bacteria and its response upon interacting with c-di-GMP, which is also associated with cell division and virulence, may constitute viable therapeutic targets for controlling microbial infections.
- It is difficult to anticipate how the selectivity of c-di-GMP will change when receptors from prokaryotes and eukaryotes are present simultaneously within the cell.
- The conditions under which the host receptor protein detects c-di-GMP need to be elucidated.
- Interactions between c-di-GMP and c-di-GMP receptors of various species have been studied in vitro using techniques such as differential radial capillary action of ligand assays, pull-down assays, and capture compound experiments [25]. These in vitro investigations should validate the binding interaction and affinity in the host system, followed by a study of the gene expression probing the response of the cell.
- High-throughput screening of natural and synthesized compounds is required to antagonize the interaction between c-di-GMP and its receptor.
- In vitro binding experiments have indicated that certain receptors also bind c-di-GMP. However, their biological functions remain unclear (Table 1) and require further in vivo studies.

## Data Availability

Not applicable.

## Abbreviations

c-di-GMP: Cyclic bis-(3', 5')-dimeric guanosine monophosphate
DGCs: Diguanylate cyclases
EPS: Exopolysaccharides
IFN: Interferon
PDEs: Phosphodiesterases
STING: Stimulators of interferon gene
